# Outpatient Antibiotic Dispensing Trends Across Canadian Provinces, 2012–2022: A Population-Based Descriptive Study

**DOI:** 10.7759/cureus.112924

**Published:** 2026-07-18

**Authors:** Wania Mohammad Akram, Ismat Ullah, Saminah Rahman

**Affiliations:** 1 Medicine, North Brampton Medical Centre, Brampton, CAN; 2 Family Medicine, North Brampton Medical Centre, Brampton, CAN; 3 Family Medicine, Saddletowne Medical Clinic, Calgary, CAN

**Keywords:** antibiotic stewardship, antimicrobial resistance, antimicrobial use, canada, interprovincial variation, longitudinal trend analysis, outpatient antibiotic prescribing, population-based surveillance, primary care

## Abstract

Background: Outpatient antibiotic prescribing represents the principal source of antimicrobial exposure in Canada and a key modifiable driver of antimicrobial resistance (AMR). Prescribing patterns vary across provinces and population groups and are influenced by stewardship initiatives as well as major system-level disruptions, including the COVID-19 pandemic.

Objective: The objective of this study is to descriptively synthesize publicly available Canadian surveillance and pharmacoepidemiologic data on outpatient antibiotic dispensing trends from 2012 to 2022, with emphasis on temporal trends, interprovincial variation, age-specific prescribing patterns, and antibiotic class distribution.

Methods: A population-based ecological descriptive study was conducted using publicly available Canadian surveillance and pharmacoepidemiologic data sources. This study was designed as a descriptive ecological synthesis of aggregated publicly available data rather than an original longitudinal analysis using individual-level or complete province-level microdata. The primary unit of analysis was the province-year, and antibiotic utilization was expressed as prescriptions per 1,000 population. Data were derived from the Public Health Agency of Canada, the Canadian Antimicrobial Resistance Surveillance System (CARSS), Statistics Canada population estimates, and the National Prescription Drug Utilization Information System (NPDUIS). Analyses were descriptive and stratified by age group and antibiotic class. Given limitations in the availability of complete longitudinal province-level microdata, formal regression and time-series analyses were not performed.

Results: Outpatient antibiotic dispensing in Canada demonstrated an overall declining trend during the early study period, reaching 642 prescriptions per 1,000 population in 2013. A pronounced reduction in prescribing occurred during the COVID-19 pandemic, with a 26.5% decrease in community antibiotic dispensing during the first eight months of 2020 compared with expected pre-pandemic levels. Substantial interprovincial variation was observed in 2019, with rates ranging from 543 to 920 prescriptions per 1,000 population across provinces, representing more than a 1.6-fold difference. Age-stratified analyses indicated persistently higher antibiotic use among older adults, whereas reductions were more pronounced among children and working-age adults. Broad-spectrum agents, including fluoroquinolones and cephalosporins, remained important contributors to outpatient antibiotic utilization.

Conclusion: Between 2012 and 2022, outpatient antibiotic use in Canada was characterized by an early-period decline, substantial and persistent interprovincial variation, consistent age-related differences, and a marked reduction associated with the COVID-19 pandemic. These findings underscore the need for sustained, regionally tailored antimicrobial stewardship strategies, with particular focus on high-prescribing provinces, older adult populations, and optimization of broad-spectrum antibiotic use. Continued surveillance is essential to guide policy and support efforts to mitigate AMR.

## Introduction

Antibiotics are among the most frequently prescribed medications in outpatient clinical practice and account for the majority of antimicrobial exposure at the population level. However, a substantial proportion of outpatient antibiotic prescriptions are issued for conditions in which antibiotics provide limited or no clinical benefit, including viral upper respiratory tract infections, acute bronchitis, and non-specific pharyngitis. Inappropriate antibiotic use contributes to increased selective pressure on bacterial populations, accelerating the emergence and spread of antimicrobial resistance (AMR), while also exposing patients to avoidable harms such as adverse drug reactions, disruption of the gut microbiome, and Clostridioides difficile infection. Consequently, outpatient antibiotic use has become a major focus of public health surveillance and antimicrobial stewardship initiatives worldwide.

AMR is recognized as a major global public health threat. In Canada, national surveillance data identify antimicrobial use (AMU) as the principal modifiable driver of resistance, emphasizing the importance of reducing unnecessary antibiotic exposure to preserve the effectiveness of existing therapies. The Canadian Antimicrobial Resistance Surveillance System (CARSS) was established to monitor patterns of AMU and resistance in humans and animals across the country. Since most human antimicrobial exposure in Canada occurs through community pharmacy dispensing, outpatient antibiotic use represents a critical component of national AMR surveillance efforts [1].

Although healthcare in Canada is publicly funded, healthcare delivery is administered at the provincial level, resulting in substantial variation in primary care access, prescribing practices, diagnostic resources, and implementation of antimicrobial stewardship programs across provinces. Differences in physician practice patterns, patient expectations, healthcare infrastructure, and population demographics may all contribute to geographic variation in outpatient antibiotic prescribing. A national pharmacoepidemiologic study examining outpatient antibiotic dispensing in 2019 demonstrated marked interprovincial differences, with prescribing rates ranging from approximately 543 prescriptions per 1,000 population in British Columbia to more than 921 prescriptions per 1,000 population in Newfoundland and Labrador [2].

Temporal trends in antibiotic prescribing are also important for evaluating the impact of stewardship interventions, evolving clinical guidelines, and major public health events. During the COVID-19 pandemic, outpatient antibiotic dispensing in Canada declined substantially, with national community prescribing rates falling by 26.5% during the first eight months of the pandemic [3]. These reductions were likely influenced by decreased transmission of respiratory infections, changes in healthcare-seeking behavior, and increased reliance on virtual care. Evaluating prescribing patterns over an extended period is therefore necessary to distinguish long-term stewardship-related changes from temporary pandemic-related disruptions.

Age-specific prescribing patterns further highlight the importance of stratified analyses. National surveillance data have shown declines in antibiotic prescribing among children and working-age adults, whereas prescribing rates among older adults remained relatively stable [1]. Older adults represent a high-use population due to greater comorbidity burden, increased healthcare utilization, and higher susceptibility to bacterial infections, particularly urinary tract and skin and soft tissue infections. In contrast, respiratory tract infections account for a larger proportion of antibiotic use in pediatric populations. These differences in infection epidemiology and healthcare utilization across age groups emphasize the importance of age-stratified analyses in informing targeted stewardship interventions.

In addition to overall prescribing volume, the distribution of antibiotic classes has important implications for AMR. Broad-spectrum agents, including fluoroquinolones and cephalosporins, exert greater ecological pressure on bacterial populations and are more strongly associated with the emergence of multidrug-resistant organisms. Monitoring trends in antibiotic class utilization therefore provides important insight into prescribing quality and the effectiveness of stewardship strategies aimed at reducing inappropriate broad-spectrum antibiotic use.

Given the importance of outpatient antibiotic use in driving antimicrobial exposure and resistance, understanding long-term prescribing trends across Canadian provinces is essential for informing stewardship policy and surveillance efforts. This study aimed to characterize national and provincial trends in outpatient antibiotic dispensing in Canada from 2012 to 2022, with particular focus on geographic variation, age-specific prescribing patterns, and differences in antibiotic class utilization.

## Materials and methods

Study design

This study was conducted as a descriptive ecological synthesis of publicly available Canadian surveillance and pharmacoepidemiologic data on outpatient antibiotic dispensing from 2012 to 2022. The primary unit of analysis was the province-year, and antibiotic utilization was reported as annual prescription dispensing rates per 1,000 population. The study aimed to characterize temporal trends, interprovincial variation, age-specific prescribing patterns, and antibiotic class distribution in outpatient settings across Canada. No individual-level patient data were analyzed, and formal longitudinal regression or interrupted time-series modeling was not performed. 

Data sources

Data were obtained from multiple publicly available Canadian surveillance and pharmacoepidemiologic sources. Early national outpatient antibiotic dispensing trends and baseline prescribing rates were derived from the Public Health Agency of Canada’s Human Antimicrobial Drug Use Report 2012/2013 [4]. Information regarding age-specific prescribing patterns and national AMU trends was obtained from reports published by the CARSS [1].

Interprovincial variation in outpatient antibiotic dispensing was assessed using a national pharmacoepidemiologic study reporting province-level outpatient antibiotic prescribing rates for 2019 [2]. Pandemic-related changes in antibiotic utilization were evaluated using a national ecological analysis examining outpatient antibiotic dispensing during the first eight months of the COVID-19 pandemic [3].

Population denominators stratified by province and age group were obtained from Statistics Canada population estimates and were used to calculate standardized prescription rates per 1,000 population [5]. Additional information regarding antibiotic class utilization and publicly funded outpatient prescription claims was obtained, where available, from the National Prescription Drug Utilization Information System (NPDUIS) administered by the Canadian Institute for Health Information [6].

Provincial health ministry and antimicrobial stewardship program reports were also reviewed to provide contextual interpretation of observed prescribing trends and stewardship initiatives.

Data source selection and harmonization

Data sources were included if they were publicly available Canadian surveillance or pharmacoepidemiologic reports containing outpatient, community, or ambulatory antibiotic prescribing or dispensing data from 2012 to 2022. Eligible sources provided national, provincial, age-stratified, or antibiotic-class-level estimates using population-based measures, such as prescriptions per 1,000 population. Reports limited to inpatient, veterinary, dental-only, non-Canadian, or non-quantitative data were excluded. When overlapping data were reported, the most recent or complete source was prioritized. Data were extracted by year, province, age group, and antibiotic class where available, and differences in reporting categories were summarized descriptively rather than statistically pooled.

Study population and definitions

The study population consisted of outpatient and community-dwelling populations across Canadian provinces between 2012 and 2022. Analyses were conducted at the province-year level and stratified by age group and antibiotic class where data were available.

Outpatient antibiotic prescriptions were defined as systemic antibacterial agents dispensed through community pharmacies and reimbursed through public or private payment mechanisms, depending on the data source. Annual outpatient antibiotic dispensing rates were calculated as the number of prescriptions dispensed per year divided by the corresponding provincial population and multiplied by 1,000.

Age stratification

Age-stratified analyses were conducted using categories consistent with national surveillance reporting frameworks: children (0-14 years), adults (15-59 years), and seniors (≥60 years) [1]. These categories were selected to reflect differences in infection epidemiology, healthcare utilization, and prescribing patterns across the life course.

Antibiotic classification

Antibiotics were categorized according to standard antimicrobial surveillance classifications, including penicillins, macrolides, cephalosporins, fluoroquinolones, tetracyclines, and sulfonamides/trimethoprim [4]. Antibiotics were additionally grouped into broad-spectrum and narrow-spectrum categories based on antimicrobial stewardship principles to evaluate prescribing patterns associated with increased ecological pressure and antimicrobial resistance risk.

Statistical analysis

Analyses were primarily descriptive. Annual outpatient antibiotic dispensing rates per 1,000 population were summarized nationally and provincially and stratified by age group and antibiotic class where data were available. Temporal trends were evaluated by examining changes in prescribing rates over time, including comparisons between pre-pandemic (2012-2019), pandemic (2020), and post-pandemic (2021-2022) periods.

Graphical analyses were used to illustrate national prescribing trends, interprovincial variation, age-specific prescribing patterns, and pandemic-related changes in antibiotic utilization using publicly reported surveillance indicators [1-4]. Because complete province-year longitudinal microdata were not publicly available for all years of the study period, analyses were limited to descriptive trend assessment. Formal regression modeling and interrupted time-series analyses were therefore not performed.

Ethical considerations

Ethics approval was not required because the study used publicly available aggregate surveillance and administrative data without individual-level identifiers.

## Results

National trends in outpatient antibiotic dispensing

Outpatient antibiotic dispensing in Canada demonstrated an overall declining trend during the early years of the study period. According to the Public Health Agency of Canada’s Human Antimicrobial Drug Use Report 2012/2013, the national outpatient antibiotic dispensing rate decreased to 642 prescriptions per 1,000 population in 2013, representing the lowest rate reported since the initiation of national surveillance [4]. Between 2012 and 2013, the national dispensing rate declined by 18 prescriptions per 1,000 population, indicating that reductions in community antibiotic use had already begun prior to the COVID-19 pandemic [4].

These early reductions established an important pre-pandemic baseline and suggested that antimicrobial stewardship initiatives, evolving clinical guidelines, and increasing awareness of AMR were already influencing prescribing behaviour during the first part of the decade.

**Figure 1:**
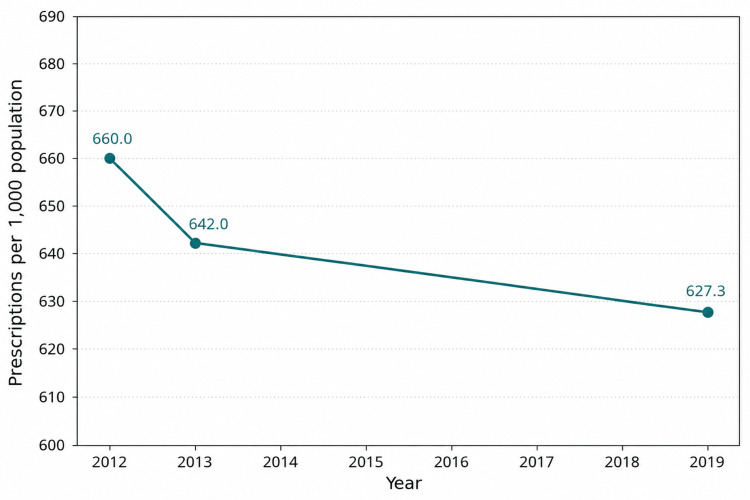
National Decline in Outpatient Antimicrobial Dispensing Rates in Canada

Figure 1 illustrates national outpatient antibiotic dispensing trends in Canada using selected surveillance anchor years.

Age-stratified prescribing patterns

Age-stratified analyses demonstrated important differences in outpatient antibiotic prescribing trends across population groups. Surveillance data from the CARSS showed that between 2010 and 2014, antibiotic prescribing decreased by approximately 8% among children aged 0-14 years and by approximately 3% among adults aged 15-59 years, whereas prescribing rates among seniors aged 60 years and older remained relatively stable [1].

These findings indicate that reductions in outpatient antibiotic use were not uniform across age groups. Although overall community antibiotic exposure declined nationally, older adults remained a high-use population throughout the surveillance period.

**Figure 2:**
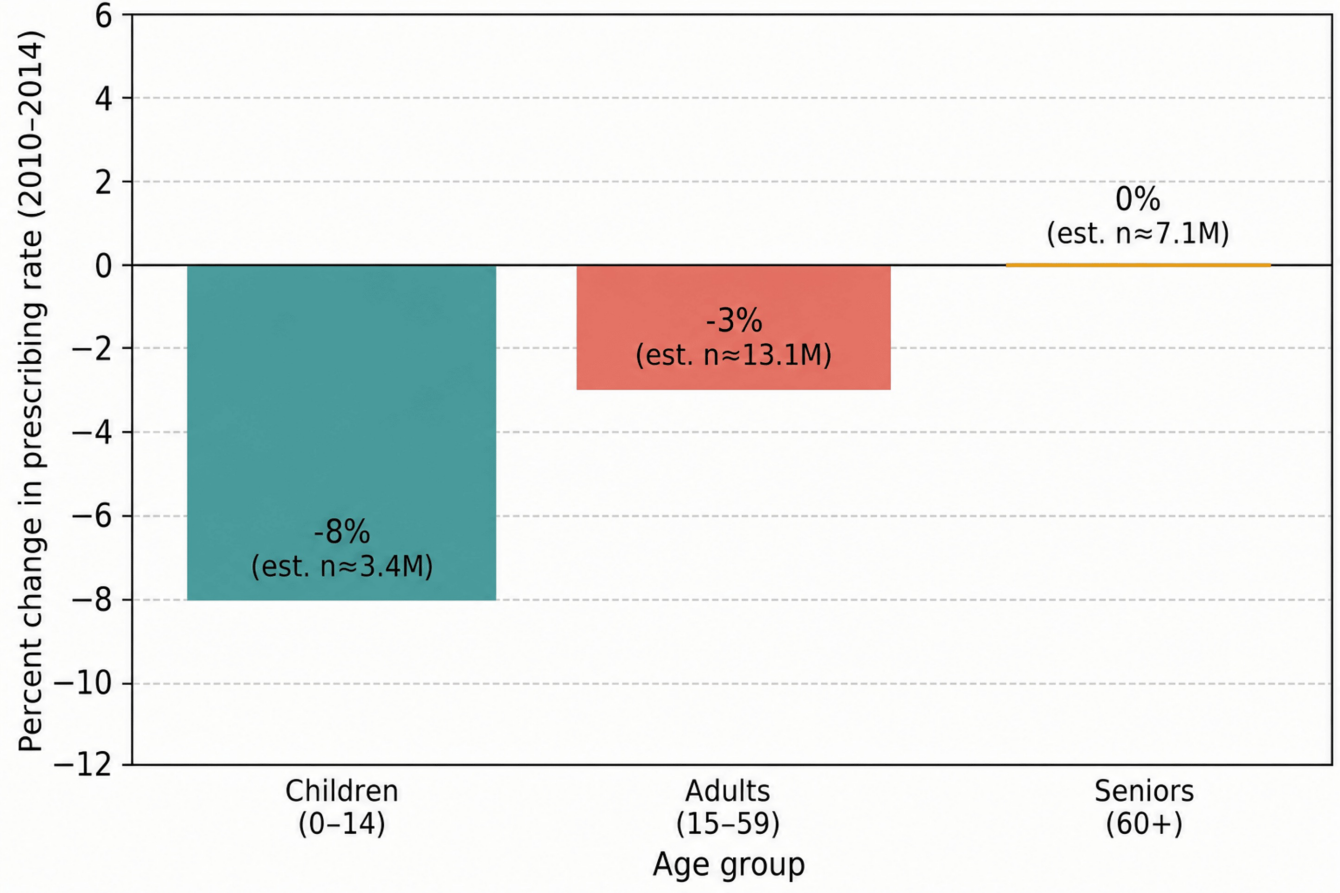
Age-Specific Changes in Antibiotic Prescribing Rates in Canada

Figure 2 displays changes in outpatient antibiotic prescribing across age groups during the early study period.

Pandemic-related changes in antibiotic dispensing

The COVID-19 pandemic was associated with a substantial disruption in outpatient antibiotic utilization across Canada. A national ecological analysis reported a 26.5% reduction in community antibiotic dispensing during the first eight months of the pandemic compared with expected pre-pandemic prescribing trends [3].

This decline represented the largest short-term reduction in outpatient antibiotic use observed during the study period. The decrease likely coincided with reductions in respiratory viral transmission, changes in healthcare-seeking behaviour, increased use of virtual care, and altered patterns of clinical encounters during the pandemic period.

Interprovincial variation in antibiotic dispensing

Substantial interprovincial variation in outpatient antibiotic dispensing was observed across Canada. In 2019, a total of 23,406,640 outpatient antibiotic prescriptions were dispensed nationally, corresponding to a crude dispensing rate of 627.3 prescriptions per 1,000 population [2].

Provincial prescribing rates varied considerably. British Columbia had the lowest outpatient antibiotic dispensing rate at 543.3 prescriptions per 1,000 population, whereas Newfoundland and Labrador had the highest rate at 920.5 prescriptions per 1,000 population [2]. Saskatchewan also exceeded the national average, with a dispensing rate of 713.7 prescriptions per 1,000 population [2]. Overall, the difference between the lowest- and highest-prescribing provinces exceeded 1.6-fold.

**Figure 3:**
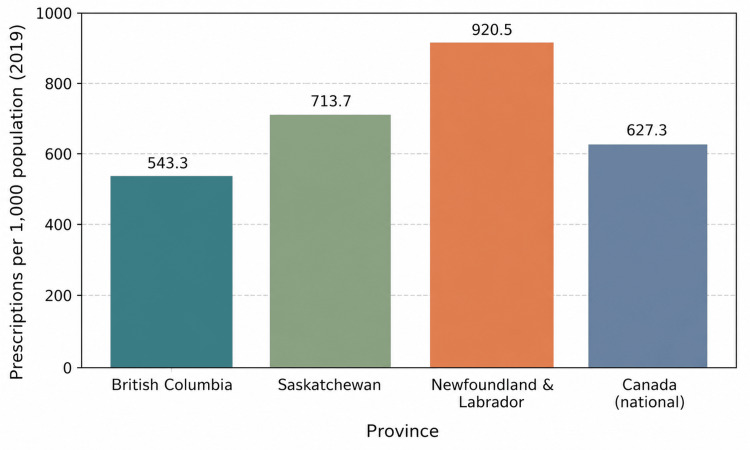
Interprovincial Variation in Outpatient Antibiotic Dispensing Rates in Canada

Figure 3 demonstrates the degree of interprovincial variation in outpatient antibiotic prescribing relative to the national average.

Antibiotic class distribution

Patterns of antibiotic class utilization varied across outpatient settings in Canada. The Human Antimicrobial Drug Use Report 2012/2013 identified amoxicillin, azithromycin, and ciprofloxacin as among the most commonly dispensed outpatient antibiotics nationally [4]. These prescribing patterns reflected the predominance of respiratory and urinary tract infection management in community practice. 

Broad-spectrum antibiotic classes, particularly fluoroquinolones and cephalosporins, represented an important component of outpatient antimicrobial exposure because of their association with AMR and Clostridioides difficile infection. Surveillance findings suggested that changes in outpatient prescribing were likely accompanied by shifts in antibiotic class utilization patterns over time [4].

Pandemic-related reductions in outpatient antibiotic use appeared to disproportionately affect agents commonly prescribed for respiratory tract infections, including macrolides and aminopenicillins [3]. However, complete province-year longitudinal data for antibiotic class-specific prescribing were not consistently available across the entire study period, limiting formal comparative analyses.

Overall trends

Taken together, national surveillance and pharmacoepidemiologic data demonstrated a gradual decline in outpatient antibiotic dispensing in Canada during the early part of the decade, followed by a major pandemic-related reduction in 2020. Despite these national declines, substantial geographic and demographic variation persisted throughout the study period. Interprovincial differences in prescribing rates remained pronounced, while age-stratified analyses showed persistently higher antibiotic use among older adults compared with younger populations.

The integration of multiple surveillance and administrative data sources provided a descriptive overview of outpatient antibiotic utilization trends across Canada between 2012 and 2022 and identified important areas for ongoing antimicrobial stewardship and surveillance efforts.

## Discussion

This study describes national and provincial trends in outpatient antibiotic dispensing in Canada between 2012 and 2022 using publicly available surveillance and pharmacoepidemiologic data sources. Overall, outpatient antibiotic use demonstrated gradual declines during the early part of the decade, substantial interprovincial variation, important age-related differences in prescribing patterns, and a major disruption during the COVID-19 pandemic. Together, these findings highlight the complexity of outpatient AMU in Canada and reinforce the importance of continued surveillance and targeted antimicrobial stewardship interventions.

National outpatient antibiotic dispensing rates declined during the early years of the study period. In 2013, the national dispensing rate fell to 642 prescriptions per 1,000 population, representing the lowest rate reported since the initiation of national surveillance and a decline of 18 prescriptions per 1,000 population compared with 2012 [4]. These findings suggest that antimicrobial stewardship initiatives, evolving prescribing guidelines, and increasing awareness of AMR were already influencing prescribing behaviour before the COVID-19 pandemic. Similar long-term reductions have also been observed in provincial studies from British Columbia following the implementation of stewardship programs and prescribing guidance updates [7].

Despite these reductions, substantial geographic variation in outpatient antibiotic use persisted across Canada. In 2019, outpatient antibiotic dispensing rates ranged from 543.3 prescriptions per 1,000 population in British Columbia to 920.5 prescriptions per 1,000 population in Newfoundland and Labrador, representing more than a 1.6-fold difference between provinces [2]. Such variation likely reflects differences in healthcare delivery, diagnostic practices, prescribing culture, patient expectations, and implementation of stewardship initiatives across jurisdictions. These findings support the need for province-specific benchmarking and regionally tailored stewardship strategies rather than a uniform national approach.

Age-stratified analyses demonstrated important differences in prescribing patterns across population groups. National surveillance data showed declining antibiotic prescribing among children and working-age adults between 2010 and 2014, whereas prescribing among older adults remained relatively stable [1]. Similar findings have been reported internationally, where older adults continue to experience disproportionately high antibiotic exposure despite reductions in younger populations [8]. Higher prescribing rates among seniors may reflect greater comorbidity burden, increased healthcare utilization, and greater susceptibility to bacterial infections. These findings suggest that stewardship interventions effective in younger populations may not translate directly to older adults and highlight the need for age-specific stewardship approaches.

The COVID-19 pandemic produced the most pronounced short-term change in outpatient antibiotic utilization observed during the study period. Community antibiotic dispensing declined by 26.5% during the first eight months of the pandemic compared with expected pre-pandemic levels [3]. Similar pandemic-related declines in outpatient antibiotic use have been reported internationally [9]. Reduced circulation of respiratory viruses, changes in healthcare-seeking behaviour, increased use of virtual care, and altered clinical practice patterns likely contributed to these reductions. The pandemic therefore represents a major structural disruption within the broader long-term trend of outpatient antibiotic prescribing in Canada.

Patterns of antibiotic class utilization also remain important from an antimicrobial stewardship perspective. Broad-spectrum antibiotics, including fluoroquinolones and cephalosporins, are associated with increased ecological pressure and greater risk of AMR and adverse outcomes [10]. Previous Canadian studies have demonstrated substantial interprovincial differences in antibiotic class-specific prescribing patterns, suggesting that reductions in total antibiotic use may not necessarily correspond to proportional reductions in broad-spectrum prescribing [10]. Continued surveillance of antibiotic class utilization is therefore important for evaluating prescribing quality and identifying stewardship priorities.

The findings of this study have important implications for clinical practice and public health policy. Because most antibiotics are prescribed in outpatient settings, particularly in primary care, province-level surveillance and benchmarking may support targeted audit-and-feedback interventions and stewardship initiatives in high-prescribing regions. The persistently high prescribing rates observed among older adults further suggest that seniors should remain a priority population for stewardship efforts [11]. In addition, ongoing monitoring of broad-spectrum antibiotic use may help identify prescribing patterns most strongly associated with AMR risk.

This study has several strengths, including the integration of multiple national surveillance and pharmacoepidemiologic data sources and the use of standardized prescribing rates to facilitate interprovincial comparisons. However, several limitations should also be acknowledged. As an ecological descriptive analysis, the study could not assess prescription appropriateness at the individual patient level or establish causal relationships. Differences in prescribing rates may also reflect variation in infection burden, healthcare utilization, and case mix that could not be fully accounted for using population-level data. In addition, because data were synthesized from multiple surveillance and administrative sources published across different time periods, inconsistencies in definitions, reporting intervals, population denominators, age-group categories, antibiotic-class groupings, and data collection methods may have affected comparability across sources. Therefore, comparisons across provinces, years, and population groups should be interpreted descriptively rather than as directly standardized estimates.

Overall, outpatient antibiotic prescribing in Canada between 2012 and 2022 was characterized by gradual stewardship-associated declines, persistent geographic variation, important age-related prescribing differences, and substantial pandemic-related disruption. Continued province-specific antimicrobial stewardship initiatives, particularly those targeting high-prescribing regions, older adults, and broad-spectrum antibiotic use, will remain essential to reducing unnecessary antimicrobial exposure and limiting the progression of AMR.

## Conclusions

This population-based descriptive study provides a comprehensive overview of outpatient antibiotic prescribing trends across Canadian provinces between 2012 and 2022. Over the study period, outpatient antibiotic use demonstrated gradual declines during the early part of the decade, substantial interprovincial variation, important age-related differences in prescribing patterns, and a major disruption associated with the COVID-19 pandemic. Reductions in antibiotic use were not uniform across provinces or age groups, with older adults consistently remaining a high-use population and provincial prescribing rates differing by more than 1.6-fold. These findings highlight the complexity of outpatient antibiotic utilization in Canada and underscore the importance of evaluating prescribing trends from both geographic and demographic perspectives.

The findings of this study have important implications for antimicrobial stewardship and primary care practice in Canada. Because most antibiotics are prescribed in outpatient settings, particularly in family medicine, targeted stewardship interventions focusing on high-prescribing provinces, older adult populations, and broad-spectrum antibiotic use may provide substantial benefits. Continued national surveillance using standardized prescribing metrics, together with province-level benchmarking and age-stratified analyses, will remain essential for promoting more appropriate antibiotic use. Strengthening and coordinating antimicrobial stewardship initiatives across provinces may help reduce unnecessary prescribing variation and support long-term efforts to limit the progression of AMR.
